# Supplementary material for: Multi‐dataset identification of innovative feature genes and molecular mechanisms in keratoconus
Source: J Cell Mol Med. 2024 Sep 19;28(18):e70079. doi: 10.1111/jcmm.70079 (PMC11412914; doi:10.1111/jcmm.70079)
Supplement: Supplementary file 2 — Material S2. [file JCMM-28-e70079-s002.zip › 7.GSEA analysis/3.DBNDD1/KEGG_PROTEIN_EXPORT.html]

Details for gene set KEGG\_PROTEIN\_EXPORT[GSEA]

|  || Dataset | DBNDD1.group.cls#h\_versus\_l |
| Phenotype | group.cls#h\_versus\_l |
| Upregulated in class | h |
| GeneSet | KEGG\_PROTEIN\_EXPORT |
| Enrichment Score (ES) | 0.2845823 |
| Normalized Enrichment Score (NES) | 0.9189086 |
| Nominal p-value | 0.5992293 |
| FDR q-value | 1.0 |
| FWER p-Value | 1.0 |
Table: GSEA Results Summary

  

Fig 1: Enrichment plot: KEGG\_PROTEIN\_EXPORT      
 Profile of the Running ES Score & Positions of GeneSet Members on the Rank Ordered List

  

| SYMBOL | TITLE | RANK IN GENE LIST | RANK METRIC SCORE | RUNNING ES | CORE ENRICHMENT || 1 | IMMP1L | na | 7537 | 0.113 | -0.0233 | Yes |
| 2 | SRP9 | na | 12105 | 0.046 | -0.0759 | Yes |
| 3 | SPCS1 | na | 15223 | 0.021 | -0.1295 | Yes |
| 4 | SEC62 | na | 15460 | 0.019 | -0.1028 | Yes |
| 5 | IMMP2L | na | 15771 | 0.017 | -0.0819 | Yes |
| 6 | SRP19 | na | 15839 | 0.017 | -0.0547 | Yes |
| 7 | SRP54 | na | 16397 | 0.013 | -0.0480 | Yes |
| 8 | SEC11A | na | 17254 | 0.008 | -0.0594 | Yes |
| 9 | SRP68 | na | 17599 | 0.006 | -0.0597 | Yes |
| 10 | SEC11C | na | 17938 | 0.003 | -0.0640 | Yes |
| 11 | SPCS2 | na | 18559 | -0.001 | -0.0796 | Yes |
| 12 | SEC61B | na | 18883 | -0.004 | -0.0829 | Yes |
| 13 | SRP14 | na | 19121 | -0.005 | -0.0804 | Yes |
| 14 | SEC61A2 | na | 19591 | -0.009 | -0.0778 | Yes |
| 15 | SEC63 | na | 20239 | -0.015 | -0.0705 | Yes |
| 16 | SEC61A1 | na | 20775 | -0.020 | -0.0518 | Yes |
| 17 | SEC61G | na | 21335 | -0.025 | -0.0248 | Yes |
| 18 | SRPRB | na | 21574 | -0.028 | 0.0158 | Yes |
| 19 | SRP72 | na | 22277 | -0.035 | 0.0553 | Yes |
| 20 | HSPA5 | na | 23780 | -0.053 | 0.1034 | Yes |
| 21 | OXA1L | na | 23915 | -0.055 | 0.1938 | Yes |
| 22 | SPCS3 | na | 24851 | -0.069 | 0.2846 | Yes |
Table: GSEA details [plain text format]

  

Fig 2: KEGG\_PROTEIN\_EXPORT      
 Blue-Pink O' Gram in the Space of the Analyzed GeneSet

  

Fig 3: KEGG\_PROTEIN\_EXPORT: Random ES distribution      
 Gene set null distribution of ES for **KEGG\_PROTEIN\_EXPORT**
